# Economic impact of new active substance status on EU payers’ budgets: example of dimethyl fumarate (Tecfidera^®^) for multiple sclerosis

**DOI:** 10.3402/jmahp.v2.23932

**Published:** 2014-03-04

**Authors:** Mondher Toumi, Guy Jadot

**Affiliations:** 1 UFR d'Odontologie, University Claude Bernard Lyon I, FR-69100, Villeurbanne, France; 2 Laboratoire « Santé, Individu, Société »-EAM 4128 Faculté de Médecine Laënnec 7-11 rue Guillaume Paradin – Bât B, Lyon, France

**Keywords:** NASs, budget impact, payers, HTA assessment, dimethyl fumarate

## Abstract

**Background:**

Recently, collaboration between regulators and payers was set up and was mainly focused on evidence generation along product clinical development. However, neither the regulatory path nor the new active substance status (NASs) was considered. Granting NASs will provide the product with 8 years of data protection and 2 years of market exclusivity during which no generic could enter the market.

**Objective:**

To review the economic impact (for payers) of NASs granted by the European Medicines Agency (EMA) for dimethyl fumarate (DMF), developed by Biogen and approved for multiple sclerosis (MS) as Tecfidera^®^ on 3 February 2014.

**Method:**

We reviewed the available DMF-containing products and identified their indication and price through relevant databases and official Web sites. The economic impact of Tecfidera^®^ on payers’ budgets was calculated assuming NASs was or was not granted. The forecast was identified in Datamonitor.

**Results:**

Results identified four products already containing DMF as the main or unique active substance. This would have potentially prevented Tecfidera^®^ from being granted NASs. The EMA Committee for Medicinal Products for Human Use (CHMP) denied Tecfidera^®^ NASs and, following a company appeal, reversed its position opening as polemic. The impact of that decision has been evaluated at €7 to €10 billion over a 10-year period.

**Conclusion:**

NASs is a critical decision because it does have a major budget impact for payers, and it prevents generic competition. Current European Union (EU) regulations on that topic are unclear and open up too many interpretations thus distorting fair trade and affecting payers’ bills. Greater clarity and more stringent rules are required to prevent mistrust of this EMA decision.

During the previous decade, there was a high level of concern from payers that the evidence required for granting a marketing authorization and how that evidence is disseminated do not help in assessing the value of newly approved drugs and in setting proper prices. In 2008 (1), the last high level forum extensively addressed how payers and regulators might collaborate to improve the situation. This led to a wide range of initiatives including various areas of collaboration between payers and regulators. These include, for example, joint scientific advice from the EMA and from health technology assessment (HTA) agencies, movement away from the requirement for the applicant to perform placebo-controlled pivotal phase III studies – historically preferred by the regulators – to conducting studies with active comparators that are preferred by HTA agencies, and expansion of the European public assessment report (EPAR) to include more information to assist in the HTA.

As a reciprocal measure, HTA agencies often have involved medicine regulators in some of their activities through early dialogue with the European Network for Health Technology Assessment (EUnetHTA). This has resulted in the publication of a joint 2013–2015 three-year working plan from the EMA and the EUnetHTA (2) outlining the areas in which the two organizations will collaborate.

However, there is little or no interaction between payers and regulators on the regulatory path either for a new medicine or in relation to the corresponding legal basis for product assessment that is selected by the applicant (i.e. whether the assessment is for a full, generic, hybrid, or similar biological, well-established use, fixed combination, informed consent applications, etc.).^1^ This is an important omission in the current approach because the clinical development program approved by the regulatory agency leads directly to a specific legal basis for assessment and can also be critical in the assessment of new active substance status (NASs) by the EMA.

Being granted NASs will allow a product to enjoy 10 years of regulatory exclusivity (8 years of data exclusivity plus 2 years of market exclusivity) with the possibility of obtaining one additional year of exclusivity.^2^ The impact on the applicant's revenue stream and on payers’ budgets could be dramatic. Decisions made by the EMA about NASs will also have critical consequences for generic companies’ ability to enter product markets and to compete with lower priced products.

Dimethyl fumarate (DMF) for multiple sclerosis (developed by Biogen IDEC, Inc., under the brand name Tecfidera^®^), has recently been the subject of a controversy as whether or not it deserved NASs. It represents a perfect example for illustrating the importance of NASs for payers.

## Objective

The aim of this article is to review the potential financial impact for payers and for society of whether or not NASs is granted by the EMA using the current example of DMF. We will review the likely financial impact on payer budgets of the NASs granted to DMF.^3^ Tecfidera's^®^ EMA marketing authorization approval for the treatment of MS is currently pending, but the EMA CHMP has indicated (on appeal by Biogen) that Tecfidera^®^ should be granted NASs (3). Tecfidera^®^ will be available in two strengths: 120 mg DMF and 240 mg DMF.

## Method

We searched for the availability and indication of DMF products in the EU through national medical agencies, health insurance, and HTA Web sites. This was complemented by literature searches to identify approved indications and common usage of DMF in EU countries.

We searched for relevant information to identify, where possible, the price of Tecfidera^®^ in the countries where it was launched. In countries where it was available, the pharmacy retail prices were identified through publicly available pricing databases and official gazette databases. In addition, we searched in company Web sites, press releases, and EU HTA Web sites in order to collect appropriate information related to the potential price of Tecfidera^®^ in the EU.

We reviewed the forecasts as provided by syndicated reports on MS (Datamonitor Healthcare, https://service.datamonitorhealthcare.com). Based on those forecasts, we aggregated the drug budget impact for payers for the five largest European markets: France, Italy, Spain, Germany, and the United Kingdom (EU5) over a 10-year period. We considered two alternative scenarios – with and without the grant of NASs to Tecfidera^®^. A sensitivity analysis was performed by varying the forecast by ±20%. We used the French generic policies as average assumptions for EU5: 1) the price discount of the branded product following generic entry is 20%; 2) the market share of generic products following launch is 80%; and 3) the price of generic competition in the market is 20% of the price of the originator product. This provided the baseline scenario.

We compared the budget impact for payers for the various scenarios but no statistical test was performed.

## Results

DMF is available in European markets in Germany, the Netherlands, and Italy. DMF-containing products are also in use on an unlicensed basis in the UK (4, 5) (Table 1). For comparison, prices of existing DMD drugs in the five largest European countries are presented in Table 2.

**Fumaderm**^®^ consists of DMF and monoethyl fumarate (MEF) salts and is marketed by Biogen IDEC, Inc. It has been available in Germany since 1994 for the treatment of psoriasis. It is available in two strengths of 30 mg of DMF and 75 mg of MEF, and 120 mg DMF and 95 mg MEF (6).

**Psorinovo**^®^ has DMF as the sole active ingredient and has been used in the Netherlands on an unlicensed basis as a pharmacy preparation for the treatment of psoriasis. GMP Apotheek Mierlo-Hout manufactures Psorinovo (7) in three strengths of 30 mg DMF, 120 mg DMF, and 240 mg DMF.

**Psocaps** is sold as a food supplement in Italy by the manufacturer Dermatica s.r.l., Padua, and is not indicated for the treatment of any medical condition (8). It is available in a single strength of 120 mg fumaric acid esters (9).

**Table I:** Price comparison (retail + VAT) of DMF as sold or proposed price in the countries where DMF is unavailable

	Indication	30 mg DMF	120 mg DMF	240 mg DMF
UK* Tecfidera^®^	Multiple Sclerosis	Not marketed in this strength	€413.32 (£343.00) per 14 pack	€1654.46 (£1,373) per 56 pack
Germany Fumaderm^®^	Psoriasis	€116,47 per 40 pack	€229.82** per 70 pack	Not marketed in this strength
The Netherlands Psorinovo^®^	Not a licensed medicinal product; made by pharmacy preparation	€59.55 per 100 pack	€89.55 per 100 pack	€170.10 per 100 pack
Italy Psocaps^®^	Not a licensed product; sold as food supplement	Not marketed in this strength	€43.00 per 60 pack	Not marketed in this strength

* The price in the UK is a wholesale selling price because the distribution of Tecfidera^®^ is most likely to be via a non-retail route; estimated based on the exchange rate of 1£=1.205€.

** Fumaderm^®^ is also available in Germany in the 120 mg strength in package sizes of 100 and 200 with corresponding retail + VAT prices of €319.47 and €624.18, respectively.

**Table II:** Price comparison (retail + VAT)* of existing disease-modifying drugs (DMD) per pack*** in € across the five largest European countries

DMD	Germany	France	Italy	Spain*	UK**
Avonex^®^	1,707.23	864.80	1,176.96	927.40	787.86
Rebif^®^ 22 µg	1,572.60	823.62	1,138.50	867.42	739.09
Rebif^®^ 44 µg	1930.94	880.80	1,530.82	1,272.04	979.65
Betaferon^®^	1,569.65	920.97	1,275.04	957.75	718.74
Extavia^®^	1,445.12	762.60	1,275.04	957.75	718.74
Copaxone^®^	1,664.51	832.04	1,206.17	870.65	619.14
Gilenya^®^	2,324.87	1923.17	2,970.92	1,722.15	1,770.87
Tysabri^®^	2,502.59	1,800.00****	2,681.09	1,760.47	1,361.28

* Hospital acquisition prices because the funding of MS treatments come from the hospital budgets.

** Wholesaler selling price due to the distribution system that is non-retail; estimated based on the exchange rate of 1£=1.205€.

*** A pack provides a supply for 28 days of treatment (with the exception of Copaxone^®^ in Germany and Betaferon^®^ and Extavia^®^, for which pack size corresponds to 30 days).

**** The price for Tysabri^®^ in France has been published in the Journal Officiel (without VAT); this is not a public price; it is exclusive to hospitals.

Sources: Lauer-Taxe (Germany), Ameli (France), Farmadati (Italy), Botplus (Spain), MIMS (UK).

Fumaric acid is sold on the Internet^4^ as a nutritional supplement in a 500 mg package.

**Fig. 1:**
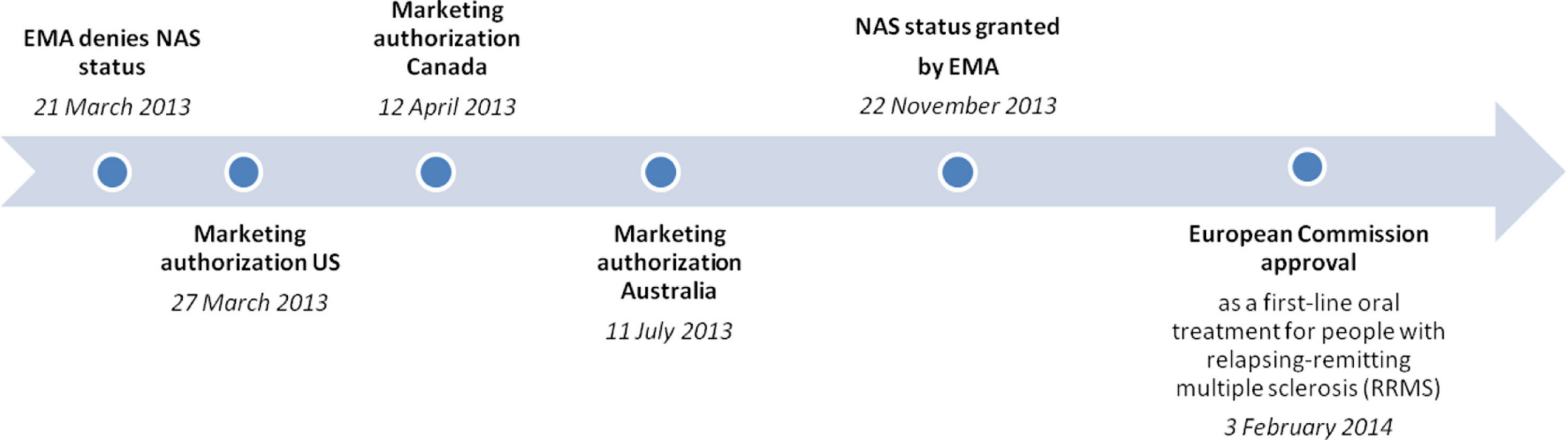
Marketing authorization process for Tecfidera^®^. EMA: European Medicines Agency; NAS: new active substance.

Tecfidera^®^ received a U.S. marketing authorization on 27 March 2013 and was launched thereafter with an annual list price of $54,900 (corresponding to around €3,350 per 56-pack of 240 mg DMF). Biogen also received a marketing authorization in Australia on 11 July 2013 (10) and launched Tecfidera^®^ with a list price of AU$1803.24 (corresponding to around €1,180 per 56-pack of 240 mg DMF, excluding VAT, which is not applied to pharmaceuticals in Australia). Tecfidera^®^ was launched in Canada on 12 April 2013 (11) and is sold with an annual list price of CAD$21,000 (corresponding to around €1,150 per 56-pack of 240 mg DMF) (Fig. 1).

### Budget impact

The forecast for Tecfidera^®^ as performed by Datamonitor Healthcare is expected to achieve sales of €700 million in 2014 and to reach peak annual sales of €1.440 billion in the EU5 by 2018. Over a 10-year period, sales are estimated to be €12 billion in the EU5.

Assuming no NASs, for the base scenario, the applicant would generate €1.5 billion while generic companies would generate €1.8 billion, making a total of €3.3 billion. This further assumes that generic competition would enter the market 1 year after Tecfidera^®^ is launched.

It therefore appears that the effect of granting NASs to Tecfidera^®^ will be a budget impact for health-care payers across the EU5 of €8.7 billion during the 10-year exclusivity period. Given the assumptions made, a reasonable estimate of the budget impact of Tecfidera's^®^ NASs grant is from €7–10 billion in the EU5 for health-care payers.

## Discussion

### Regulatory aspect

Obviously NASs is a critical issue from the perspectives of the applicant, the generic competition, and of the payers. NASs has, to date, been overlooked by payers despite their increased scrutiny of drug repositioning. Drug repositioning is a way to provide new benefit from an existing product in a new indication or geography. It is an efficient and low-risk development strategy for pharmaceutical companies and has contributed to numerous new products. For example, memantine, an old German non-specific psychotropic drug, was repositioned by Merz for Alzheimer's disease. It has been the only approved drug for many years for patients with severe Alzheimer's and has reached a blockbuster status (12–14). This was an outstanding contribution for Alzheimer's disease management.

Since 2011, the CHMP has adopted the practice of assessing whether or not a product will qualify for NASs during the assessment of its benefit/risk profile. Obtaining NASs is critical for the applicant because it allows access to the centralized procedure at the EMA; second, it provides the product with 10 years of exclusivity; and third, most member states’ HTA agencies would not permit premium pricing for products without NASs.

In practice, if a substance has been previously authorized as a medicinal product in the EU, it should not qualify for NASs. To assist it in assessing NASs, the CHMP uses the EU Commission's guidance as set out in the Notice to Applicants. Annex 1 of Chapter 1 of that document^5^ has a non-exhaustive list of compounds that could qualify as NASs. Annex 1 is not a legally binding document, but it provides the CHMP with guidance on how to apply the relevant legislation.

Article 10(2)(b) of Directive 2001/83^6^ is the key primary legislative source that sets out the legislator's intent in relation to the grant of NASs. This provision does not mirror Annex 1 in the Notice to Applicants and does not take the same stepwise approach. It is the ‘opposite’ of the Annex in the Notice to Applicants, defining what can be considered to be the ‘same active substance’ in the context of abridged applications. Further, Article 1(3a) of Directive 2001/83^7^ provides a definition of ‘active substance’. Presumably, what is considered to be the ‘same active substance’ under Article 10(2)(b) cannot also qualify for NASs.

The EMA has recognized the need to provide guidance to applicants seeking to establish NASs for line extension products. In 2012, the agency produced a reflection paper (15) setting out the data it expected would accompany claims by applicants that a product should be granted NASs. This reflection paper indicated that, although in some circumstances convincing non-clinical data may establish the difference in safety and efficacy required to take a line extension product outside Article 10(2)(b), the agency would usually require comparative clinical data.

The burden of proving the clinical differentiation in order to establish NASs for line extension products lies with the applicant. It is up to the applicant to generate and submit for assessment evidence of the clinical safety and also any efficacy differentiation versus the earlier comparator product. The value of any preclinical proxy or surrogate is often questionable when claiming the better safety of a product. For example, in the field of MS, the success story of natalizumab, once thought to be a revolution (due to high efficacy) in the management of MS, was halted due to life threatening safety issues (progressive multifocal leukoencephalopathy). This highlights the fact that it remains very difficult to claim a good safety profile or a better safety profile for a new product until it has been used in large patient populations. Until then, any properties extrapolated to humans from non-clinical or clinical studies remain speculative. This then places the agencies in difficult positions, such as is the case for Tecfidera^®^ where Biogen has not published any comparative clinical data suggesting any properties superior to Fumaderm^®^.

With regard to Tecfidera^®^, the position of the CHMP has not been straightforward. The initial opinion was to deny NASs to Tecfidera^®^ on 21 March 2013 (3). However, the CHMP also provided a positive opinion for an EU approval at that time (i.e. it concluded that the benefit-risk assessment of the product was positive and it recommended that a marketing authorization be granted). The applicant, Biogen, lacking the commercially important positive ruling on NASs, delayed the launch of Tecfidera^®^ in the EU until the CHMP reviewed its position. On 22 November 2013, the CHMP changed its opinion on NASs and granted Tecfidera^®^ NASs. Biogen's strategy to delay the launch of the product might have weighed on the CHMP when it was making its November decision to grant NASs to Tecfidera^®^, but this ultimately proved successful for the applicant. Indeed, some analysts have suggested that the Biogen's threat to not launch Tecfidera^®^ allowed it to obtain NASs ‘which [in their opinion] it does not deserve’ (16) (Figure 1).

In November 2013, the CHMP announced that, in its opinion, Tecfidera^®^ should be granted NASs because it was ‘different’ from Biogen's old psoriasis product, Fumaderm^®^. The only explanation given was that: ‘Fumaderm is composed of DMF, calcium salt of ethyl fumarate, magnesium salt of ethyl hydrogen fumarate, and zinc salt of ethyl hydrogen fumarate’ (15).

It is difficult to determine which of the component substances in Fumaderm^®^ is active. In this context, the CHMP had a problem – how could Tecfidera^®^ be differentiated from Fumaderm^®^? Biogen claimed, without data or other evidence, that MEF might be responsible for certain Fumaderm^®^ safety properties (17), but this brief commentary is significantly different from the comparative clinical data required by the 2012 EMA Reflection Paper. A significant body of non-clinical and clinical evidence suggests that the MEF salts have no effect on the activity of Fumaderm^®^ (18–20). Current EU psoriasis guidelines also state that the active ingredient in Fumaderm^®^ is DMF (21). So given the doubt cast on MEF's role in Fumaderm^®^, it seems all the more inconsistent with the publicly available evidence that CHMP should consider that the lack of MEF in Tecfidera^®^ provides the ground for this product's grant of NASs.

Under the clear definitions contained in Directive 2001/83,^8^ a substance can only be an ‘active substance’ if it exerts some effect on the action of the product. The evidence suggests that MEF does no such thing; it therefore appears that the CHMP may have been premature in its finding that Fumaderm^®^ and Tecfidera^®^ are ‘different’ in their active substance composition.

Hence this is perhaps a remarkable decision, suggesting to future applicants how to use old products. Usually later products only qualify for NASs if they differ significantly from earlier products with regard to clinical safety and efficacy. Because Tecfidera^®^ targeted a new indication, a head-to-head clinical comparison of this product with Fumaderm^®^ was not feasible. The CHMP decision on NASs for Tecfidera^®^ is based on the removal of an inactive component of Fumaderm^®^. In the context of the published literature indicating MEF plays no role in the activity of Fumaderm^®^, the 2012 EMA Reflection Paper online extension products, and the clear definitions contained in Directive 2001/83, one would have thought that the CHMP would ask Biogen to prove its claims that Tecfidera^®^ was clinically deserving of NASs. The fact that it did not could provide a useful new route to NASs for future applicants seeking to repurpose or reformulate old products.

### Distortion of competition

By granting NASs to Tecfidera^®^, the CHMP has provided Biogen with a robust competitive position and has made it impossible for any generic company to enter the EU market with a mono-DMF product. The limited clinical evidence on which the decision was based raises significant issues surrounding the distortion of competition. Indeed, the 10 years of exclusivity related to NASs is not open for direct legal challenge (unlike as with other market exclusivity rights such as patents). In order to challenge data exclusivity, a competitor's company must go to the expense of developing its own rival product, submitting it for assessment, and then suing the relevant regulatory agency for judicial review when the application is rejected. However, putting aside its value as a barrier to the entry of generic competition, the CHMP's grant of NASs is potentially far more advantageous to Biogen in member state pricing and in reimbursement systems.

Providing an example of current competitor products in development, the Dutch company Synthon has a product in development and has filed patents on a new DMF formulation that it has developed to bring that product to the market (22). The NASs grant for Tecfidera^®^ is likely to materially impact Synthon's ability to use the abridged procedure for its product.

Although the NASs will have an important favorable impact on Biogen's revenue, it will have a major unfavorable impact on Synthon. Assuming that Synthon is as innovative with this active substance as that of Biogen (both companies have taken old products and reformulated them), why should Biogen now receive a de facto 10-year monopoly on the product and Synthon be barred from EU market entry for 10 years? Non-transparent CHMP decisions open to debate their legitimacy to grant NASs.

The CHMP decision to grant NASs to Tecfidera^®^ does not seem to be based on current regulations in force, is lacking transparency, and thus seems to be a barrier to fair competition. It is likely that more companies are preparing various improved DMF formulations in anticipation of the fact that they will compete with Tecfidera^®^. The NASs decision is likely to lead to future legal disputes as companies challenge the exclusivity granted by the CHMP that, at face value, appears unsupported both by published literature and the test of Directive 2001/83.

### Incentives to reward innovation

The failure to obtain NASs does not mean companies are precluded from protecting their investment in innovation. Products can also be protected by patents (and usually are). A patent provides its owner with an exclusive right to exploit the product covered by the patent for 20 years (which can be extended for up to an additional 5 years in the case of pharmaceutical patents by applying for a supplementary protection certificate). Biogen, for example, has been granted one patent (23) potentially covering Tecfidera^®^ and has a number of other patent applications, which may cover Tecfidera^®^, that are pending. Although one could read Biogen's unwillingness to launch Tecfidera^®^ without NASs as an admission of the vulnerability of these patents, it is difficult to know whether this really contributed to Biogen's decision to appeal NASs in early 2013.

### Impact on payers’ drug budget and allow patient's access

Because the potential budgetary impact of granting NASs to a product is likely to be long-lasting and dramatic for payers, such decisions should be taken carefully through a robust and transparent assessment procedure. The current 2012 EMA Reflection Paper developed by the CHMP provides some guidance whether the NASs of obvious line extension products should be assessed by the EMA. However, it does not clearly specify how repositioned products should be considered when accompanied by a change in indication such that no comparative clinical studies are possible.

According to Murteira et al. Tecfidera^®^ took the following path: repositioning within the same company, marketing authorization granted, patent expiry, a different brand name, through serendipity, on target, and within a different therapeutic area (12).

It is predicted that Tecfidera^®^ will become a dominant MS therapy and will reach a global peak-year sale of €4.5 billion (24). Therefore, our estimate of €12 billion sales over 10 years in the EU5 and a value of NASs of €7–10 billion looks realistic.

Biogen initially developed Tecfidera^®^ for severe psoriasis patients, and two clinical trials were conducted including a phase II dose finding study and a large phase III study. The dossier seems to have been considered to be compelling because Biogen filed an application for this indication in Germany. It was then withdrawn on the request of the applicant (25), and Tecfidera^®^ was positioned for MS. This suggests a potential strategy on the part of Biogen that first approving Tecfidera^®^ for MS would assist in gaining NASs and would prevent early generic mono-DMF product competition in the field of psoriasis. The different indication pursued by Biogen has been instrumental in justifying the lack of a head-to-head trial. This strategy has proved successful because Biogen did eventually obtain the CHMP decision to grant NASs to Tecfidera^®^.

Although this has proved an effective strategy for obtaining NASs for Tecfidera^®^, it has denied severe psoriasis patients access to a new and effective therapy. The phase III trial for Tecfidera^®^ in psoriasis showed a reduction of the primary outcome psoriasis area and severity index (PASI) of 68% for Tecfidera^®^ versus 10% for placebo (25). The severe psoriasis patient indication might also be later considered as a life cycle management option for Tecfidera^®^, possibly adding an additional year's exclusivity to its current 10 years – by utilizing Article 10(1) Directive 2001/83.^9^ We recognize that companies need to consider benefit and risk on return on investment when making decisions and developing product strategies. This is legitimate and should not be challenged. However, regulators should be aware of such strategies when making decisions such as that of NASs for Tecfidera^®^.

Considering that the applicant withdrew the Tecfidera^®^ psoriasis file in Germany in 2007 and delayed the launch of Tecfidera^®^ for MS until the NASs was resolved by CHMP, it is unclear if they would have withdrawn the application if the CHMP had again denied NASs on appeal. The company stated that the NASs provide it with reassurance for the return on the large investment undertaken by the company (3). What is clear is that if Biogen had withdrawn the Tecfidera^®^ dossier from the EMA, there would have been no marketing authorization and no opportunity for generic competition in MS. It is unclear how heavily this threat might have figured in CHMP's deliberations when granting NASs.

## Conclusion

Increased attention should be given to the process of granting NASs because it has a significant impact on the length of exclusivity that a medicinal product may obtain; and therefore, it affects the ability for competitors to come to the market and offer cheaper generic alternatives to compete with premium priced products. On the other hand, it does have a major impact on an applicant's opportunities for return on investment. The value of these decisions, and their costs to EU health-care budgets, runs in the billions of euros.

In 2012, the CHMP Reflection Paper clarified some questions concerning the more usual line extension products encountered, but major gray areas remain open to interpretation. Therefore, there is still the possibility of divergent interpretations and a lack of reliability and predictability in such decisions. Using Tecfidera^®^ as an example: Biogen repurposed an old product by removing a component that many publications assumed to be inactive. A full clinical development program was then conducted in a new indication, and no comparative clinical study was generated or submitted to the EMA. Current guidance and regulatory practice seems incapable of properly dealing with this situation. However, we suggest the answer should be clear. When the applicant seeks NASs, the burden of proof should be on the applicant to demonstrate that the product really is something new.

NASs should not be considered as an incentive for innovation for applicants. There are serious ethical questions to be addressed if regulators encourage full clinical dossiers and extensive patient testing of products where the applicant might have been able to take a less trial-intensive route to its marketing authorization. Patents are, and should remain, the reward for invention and are a distinct incentive for NASs. The consequences on the drug budget for payers could be dramatic if the current legislation is to be interpreted as encouraging the repurposing of old products to obtain NASs. The responsibility of the CHMP in making such decisions is weighty indeed and should be highly transparent. Currently, it is not. To further ensure robust and reliable decisions are made, technical appeal tribunals should exist to review the CHMP and commission decisions in this area, not only for the applicant but also for other companies affected by NASs decisions, as well as public and private payers in the EU.

If the current regulation is found to be inappropriate, it should not be bypassed under the excuse of providing patients access. Rather, it should be revised to reflect on how to properly deal with this matter. The European Commission should carefully consider the appropriateness of current regulations in order to allow patient access, fair competition, and to incentivize innovation emergence.

Furthermore, payers should consider putting the procedures for granting NASs on the table for future collaborations with regulators. In the past, interactions between payers and regulators have been restricted to evidence sharing; interactions between these entities should be broadened. Given that decisions made by the CHMP such as that granting Tecfidera's^®^ NASs cost EU payers many billions of euros, there is no doubt that NASs will become a hot topic for payers in the near future.

## Footnotes

1 European Union. Directive 2001/83/EC of the European Parliament and of the Council of 6 November 2001 on the Community Code relating to Medicinal Products for Human Use (as amended). Article 8 and 10 OJ L 311, 28.11.2001, p. 67.

2 European Union. Directive 2001/83/EC of the European Parliament and of the Council of 6 November 2001 on the Community Code relating to Medicinal Products for Human Use (as amended). Article 10. Available from: http://ec.europa.eu/health/files/eudralex/vol-1/dir_2001_83_consol_2012/dir_2001_83_cons_2012_en.pdf.

3 We have used the brand name Tecfidera^®^ in this article to avoid confusion. DMF is contained in many different products that are discussed in this article so when referring to a specific product we will use the brand name.

4 http://www.super-smart.eu/article.pl?id=0260&lang=fr&fromid=GG132&gclid=CMWV6aiQtbwCFSYTwwodOQgAdA.

5 European Commission. EudraLex – Volume 2 – Pharmaceutical Legislation Notice to applicants and regulatory guidelines medicinal products for human use. [Online]. Available from: http://ec.europa.eu/health/documents/eudralex/vol-2/index_en.htm [Accessed 27 January 2014].

6 European Union. Directive 2001/83/EC of the European Parliament and of the Council of 6 November 2001 on the Community Code relating to Medicinal Products for Human Use (as amended). Article 10(2)(b). OJ L 311, 28.11.2001, p. 67.

7 European Union. Directive 2001/83/EC of the European Parliament and of the Council of 6 November 2001 on the Community Code relating to Medicinal Products for Human Use (as amended). Article 1(3a). OJ L 311, 28.11.2001, p. 67.

8 European Union. Directive 2001/83/EC of the European Parliament and of the Council of 6 November 2001 on the Community Code relating to Medicinal Products for Human Use (as amended). Article 1(3a). OJ L 311, 28.11.2001, p. 67.

9 European Union. Directive 2001/83/EC of the European Parliament and of the Council of 6 November 2001 on the Community Code relating to Medicinal Products for Human Use (as amended). Article 10(1). Directive 2001/83/EC (as amended) OJ L 311, 28.11.2001, p. 67.
